# An ultra-sensitive biophysical risk assessment of light effect on skin cells

**DOI:** 10.18632/oncotarget.18136

**Published:** 2017-05-24

**Authors:** Devasier Bennet, Buddolla Viswanath, Sanghyo Kim, Jeong Ho An

**Affiliations:** ^1^ Department of Bionanotechnology, Gachon University, Seongnam, Gyeonggi-Do 461-701, Republic of Korea; ^2^ Department of Polymer Science & Engineering, Sungkyunkwan University, Suwon, Gyeonggi-Do 440-146, Republic of Korea

**Keywords:** biophysics of organization, biomechanical changes, color light radiation, extracellular matrix degeneration, AFM and ECIS based analysis

## Abstract

The aim of this study was to analyze photo-dynamic and photo-pathology changes of different color light radiations on human adult skin cells. We used a real-time biophysical and biomechanics monitoring system for light-induced cellular changes in an *in vitro* model to find mechanisms of the initial and continuous degenerative process. Cells were exposed to intermittent, mild and intense (1-180 min) light with On/Off cycles, using blue, green, red and white light. Cellular ultra-structural changes, damages, and ECM impair function were evaluated by up/down-regulation of biophysical, biomechanical and biochemical properties. All cells exposed to different color light radiation showed significant changes in a time-dependent manner. Particularly, cell growth, stiffness, roughness, cytoskeletal integrity and ECM proteins of the human dermal fibroblasts-adult (HDF-a) cells showed highest alteration, followed by human epidermal keratinocytes-adult (HEK-a) cells and human epidermal melanocytes-adult (HEM-a) cells. Such changes might impede the normal cellular functions. Overall, the obtained results identify a new insight that may contribute to premature aging, and causes it to look aged in younger people. Moreover, these results advance our understanding of the different color light-induced degenerative process and help the development of new therapeutic strategies.

## INTRODUCTION

The human integumentary system is the only organ directly exposed to greater levels of light radiation from natural and artificial light environments. Natural and artificial lights are essential for daily lives; but, they cause many acute and chronic diseases and disorders, including photoaging, immunosuppression [1], ROS generation [2], skin damage [3–5] and especially carcinogenesis [6]. Environmental and lifestyle factors can play a significant role in the aging of the skin and various skin and ocular diseases, including the development of premature aging and various cancers [7–12]. Particularly, younger and lightly pigmented people are more prone to photoaging than those with dark pigmented skin [13]. Photoaging may cause fine lines, fine and coarse wrinkles, frown lines, deep wrinkles, blotchy depigmentation, increased fragility, and rough skin texture [14, 15]. Visible light (400–700 nm, 65 J cm^-2^) and sunlight radiations (6 J cm^-2^) with an initial dose produce free radicals in the cells [2, 5, 16] followed by major changes in skin cells, cell membrane damage, and collagen and the surrounding extracellular matrix (ECM) protein degenerations [5, 17], and cutaneous degradation [3].

Normally, physiologic response protects the skin from photo-damaging. The skin is composed of a wide range of phenotypic cells, including melanocytes, keratinocytes, and fibroblasts, and these play a highly important physiological and biological regulation through cells, surrounding extracellular matrix (ECM) proteins and pigments, which is mainly responsible for providing a vast physical barrier [13, 18–20] and a unique defense system against harmful environments. Furthermore, cells contain several kinds of light absorbing pigments; the biological chromophore in the skin cells may absorb a different wavelength of light, which depends on the specific kinds of biological chromophore present in the cells/tissues [21, 22]. Moreover, biomolecules including nucleic acids, carrier molecules, heme, cytochromes quinones, flavins, and eumelanin are acted as light absorbents [23, 24].

Mechanisms of photosensitization triggered by light absorbents under the blue, red, green and white light radiation are not well understood. The most common culprit is natural and artificial light radiation [2, 22], which causes free radical formation and may lead to major changes in skin cell architecture, DNA, collagen and surrounding molecules [16]. Collagens are the most abundant protein in the ECM. ECM can serve many functions, such as providing support, cell attachment, cell-to-cell communication, and differentiation. Even though disruption of the normal architecture of cells/tissue is one of the main reasons of impairing normal skin cell function and skin aging, the detailed process of impairing skin functions remains unknown. Type I collagen is the main structural protein in the dermal extracellular matrix [25, 26] which produced by dermal fibroblasts. Collagen fibrils are responsible for the durability, physical and mechanical properties of cells/tissues with improved strength. A single exposure ultraviolet light radiation can cause damage to collagen through a breakdown, disorganization, and suppression of biosynthesis, and results in loss of collagen content in the skin [27]. However, there is very little information about the relationship between visible light induced photo-ageing and mechanical properties of cells. Daily use of various light sources may also be one of the culprits for photoaging; hence, it is important to know the inferences of light radiations with different wavelength on the human integumentary system. So, the specific risks of an individual band of light such as blue, red, green and white light radiation on cells need to be analyzed more deeply for the biophysics and biomechanics of cellular viability, ultra-structural changes, degenerative process and ECM degeneration process in a real-time manner. The different color light-induced risk factor assessments in real-time biophysics and biomechanics measurement are not well established so far. So it is important to understand the cellular biomechanics and cell physiological behavior of living cell/tissue functioning upon light exposure, we choose to study the biophysical and the biomechanics behavior of human epidermal keratinocytes-adult (HEK-a); human epidermal melanocytes-adult (HEM-a) and human dermal fibroblasts-adult (HDF-a); which are believed to the responsible for the regulation and function of various primary and secondary pathway's regulations in the cells. These cells are the experimental targeted cells for this acute and chronic blue, red, green and white light radiation exposed to damage in an *in vitro* model.

The aim of this study was to provide a detailed analysis regarding the effects of different color light radiations on HEKa, HEMa, and HDFa cell monolayers. As a light source, LED light, including blue-460 nm, green-530 nm, red-625 nm and white light radiation, is employed and their toxicities were examined in real time manner. By employing multiple real-time observations, including ECIS, Bio-AFM, and FACS, it is attempted to understand the mechanism by observing biophysical and biomechanics variations in three types of cells. Moreover, this study attempted to elucidate the ECM protein degradation process and to monitor how cell functions have been deregulated. So these experiments may reveal new aspects of the photo-aged skin and age-related degradation of the skin cells.

## RESULTS AND DISCUSSION

### Impedance monitoring of the cellular response to different color light effects

**Figure 1:**
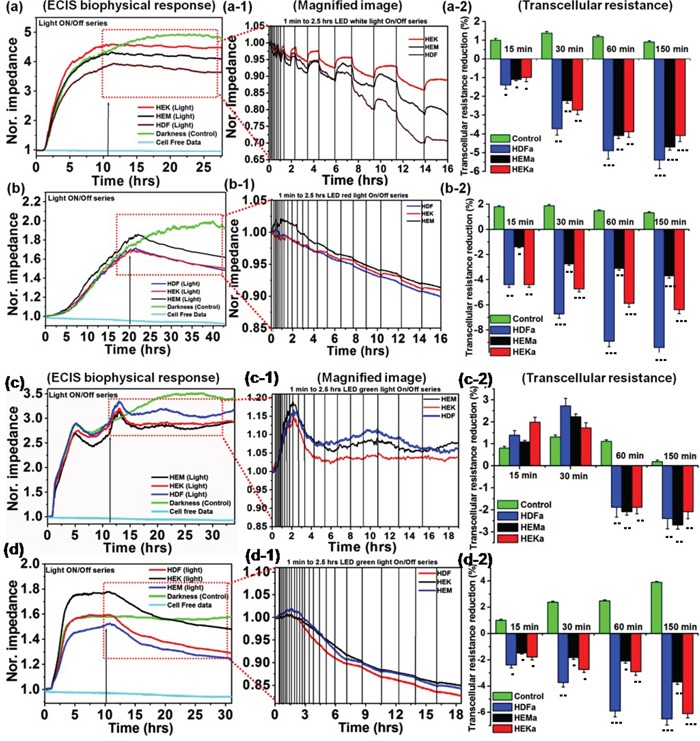
Normalized impedance profile of skin cells upon intermittent color light (white, red, green and blue) exposure, including photo-toxicity **(a-d)** Responses of different cell lines from skin tissues to elevated white, red, green and blue light sensitivity reactions are shown respectively. (**a-1** and **b-1**) Magnified image of (**a** and **b**) showing the effects of intermittent white and red-light exposure on cells, respectively. (**a-2** and **b-2**) Effect of white and red light-induced transcellular resistance, the light exposure induces a persistent reduction in resistance, but not in control experiments. (**c-1** and **d-1**) Magnified image of ‘c’ and‘d’ showing the effects of intermittent green and blue-light exposure on cell responses, respectively. (**c-2** and **d-2)** Effect of green and blue light-induced transcellular resistance, the light exposure induces a persistent reduction in resistance, but not in darkness. The light-induced transcellular resistance was compared with control (darkness) with the level of significance set at ***P < 0.0005, **P < 0.005 and *P < 0.05.

The bioimpedance setup was established (Supplementary Figure 1) to study the artificial light effect on HEKa, HEMa, and HDFa cells. Different artificial color lights (red, blue, green and white) were used, which is characterized by a spectroradiometer (Supplementary Figure 2a) and electromagnetic spectrum of each color light show within a visible region. The light-induced changes on cells were monitored over intermittent ON/Off cycle for 1-150. A normalized impedance curve is shown in Figure 1. Data are expressed as differences in normalized impedance values between control cells (darkness) and light exposed cells. The cell-free investigation, the impedance was stable for throughout experiments; which means that there is no disturbance from any external factors. The skin cell investigation, cells are attached to the detecting electrode and the resistance of the cell covered electrode increased rapidly, and then about 10-15 hours it reached steady state. After reaching the cell confluence, the lights were exposed to an on/off series with gradually increasing time intervals (1 to 2.5 hrs). The impedance value has been changed considerably in light exposed cell covered electrode, which indicates that the cells function has been altered. But the cells exposed to darkness show no changes in impedance value.

**Figure 2:**
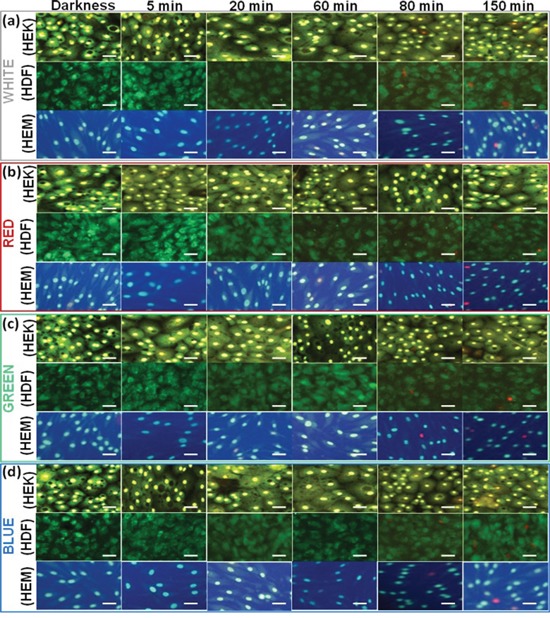
Live–Dead Cell Staining results of different cell lines upon exposure to different color light The fluorescence microscopy images were taken at 15, 30, 60, 80 and 150 min time points upon exposure to different color light. The darkness cell also imaged. Red staining indicates dead and green indicate live cells. Scale bars = 50 μm.

During initial light exposure, no changes were observed in any samples, but after 15-30 min of light exposure cycle, all cells began to show changes in biophysical impedance, which means that the reduction response was observed. The individual light exposure data, Figure 1(a) show that the normalized impedance value of white light-induced changes in different skin cells. Figure 1(a-1) compares the effect of intermittent white light exposure on various cells, control cells (darkness) and blank (cell-free) sample. At the beginning of the On/Off light exposure, the impedance value decreased sharply and increased slowly from 30 min onwards, followed by a continuous decrease of impedance with smaller scale fluctuations. At the end of 2.5 hrs light exposure cycle, the normal cellular activity, and function were impeded which means that the cell-substrate adhesion strength has been diminished. But the cell viability staining image shows that the most of the cells are viable. The white-light exposure has affected the cells in a time-dependent manner. The on/off light exposure cycle resulted in the decline/recovery of the impedance value which indicate either structural integrity breakdown or cell adhesion protein breakdown at the cell confluence. This breakdown is probably weak because the cell structural integrity and cytoskeleton have been renewed with restored impedance during the unexposed time. Figure 1(a-2) shows that white light-induced transcellular resistance reduction, which clearly depicted the comparative effect different cell cytoskeletal electrical resistance decline with the time dependency manner. Cells kept in the dark did not affect the cellular resistance, and they remained in a steady growth peak. HDFa cells show highest reduction in transcellular resistance when compared to HEMa and HEKa. In the cell viability staining HDFa cells started to die after 80 min of intermittent white-light of exposure (Figure 2(a)). On the other hand, HEK and HEMa cells survived till the 150 min intermittent white light exposure.

Figure 1(b) showed the red light-induced changes in different skin cells. Figure 1(b-1) shows the magnified image for the comparative effect of different intermittent exposure time on different cellular responses. During each cycle, the impedance value decreased slowly, and it starts from 30 min to 180 min in a time-dependent manner. As the light exposure increased, the impedance value continuously decreased. But the recovery is very less than the initial value. At the end of 2.5 hrs light exposure cycle, the normal cellular activity, and function were impeded, but the represented live/dead staining image shows that they are surviving and tolerated. The continuous impedance reductions induced by red-light were confirmed with transcellular resistance reduction Figure 1(b-2), showing a continuous decrease in the value of normalized impedance. The percentage reduction in transcellular resistance induced by red-light exposure is significantly higher than that of cells kept in darkness. The percentage of red-light-induced transcellular resistance reduction was less compared to that of white-light exposure. Moreover, the growth reduction results were confirmed with cell viability staining which shows that the HDFa cells are started to die after 80 min when cells are exposed to red light using an on/off cycle(Figure 2(b)), followed by other cells. When the cells were exposed to green light (Figure 1(c)), the impedance value increased to above-normal growth value of the initial exposure (from 0 – 30 min). After 30 min, the impedance was started to decrease very slowly. However, the continuous on/off cycle of green light exposure, the normal cellular functions were impeded. After 60 to 150 min exposure, the impedance value decreased to the lower than the normal range. The increases of impedance responses were associated with the increase of the cell-substrate coverage or increase cell-substrate binding efficiency or cell inflammation which can increase the resistance. Moreover, the increases of impedance responses may associate with the decrease of photoabsorption or photo-toleration capacity of the cells. This particular wavelength of light may have lower absorption ability of the biological chromosphere presented in the cell components. That may be one of the reasons for an initial increment of impedance value. But followed by the continuous exposure the impedance value has been decreased, which may relate to higher absorption reaction. The higher light absorption may lead to photochemical reaction, which can convert the exogenous chromophore into photoproducts that can initiate cellular changes [27]. Followed by the continuous light exposure cycle, the photosensitized chromophore molecules cause photoaddition (addition reaction that takes place under the influence of light) and photoxidation reaction, which are responsible for inducing free radicals [28], and leading to oxidative stress and phototoxicity. This type of oxidative stress effect shows in all wavelengths, but cells show higher tolerance to green light. If the cells can tolerate the specific dose of light exposure, they have the ability to show higher proliferation rate than that of cells kept in darkness. These observations are further confirmed by transcellular resistance reduction and cell viability staining (Figures 1(c-2) and 2(c)). And these results can be correlated with the biophysical response from ECIS. Finally, the ECIS analysis of the blue-light exposure on different skin cell lines was summarized in Figure 1(d). The blue light's effects were nearly similar to those of red light. However, the blue light exposure shows the most rapid impedance drop among various light exposures (Figure 1(d-1)). Here, the blue light-induced stress is continued until the 2.5 hrs, and it is also reflected in the initial rapid onset of stress by blue light. The early stages of cellular physical responses were likely not caused by apoptosis or necrosis and hence cannot be analyzed by cell viability staining. Therefore, the initial cell status is believed to be the result of light-induced stress reactions, which affects the cell-cell binding or cell-substrate binding efficiency.

Furthermore, this early stage cellular ECIS response may be responsible for the initial stage of inducing the aging process, which has been found in skin dermal cells light exposures of various wavelengths. The early decline in impedance values symbolizes the existence of the photoreaction, resulting in the increment in ROS levels [3], oxidative stress, deregulates of cellular integrity [4, 5], which ultimately loosening of cell attachment strength. This light-induced cellular reduction was also confirmed by transcellular resistance reduction and live/dead staining imaging (Figures 1(d-2) and 2(d)) and the results were correlated with the biophysical response from ECIS. The impedance of all light exposed cells obviously decreased during the photodynamic reaction, and the range of decrease is depended on the kind of light and exposure time [29]. The light absorption molecules also directly affect the photodynamic reaction by the photosensitization process, which leads to photo-induced reactions and cell damage [30]. The level of reaction is based on the chromophores content in the individual cells [31]. Therefore, in this study, we analyzed the light-absorbing chromophores density in each HDFa, HEMa and HEKa cells for the comparison of photosensitization molecules, which can give the direct understanding of photodynamic reaction by photosensitization molecules. The pigment density differences in each cell type were analyzed by UV-Visible spectroscopy and were shown in Supplementary Figure 2b. Equal quantities of HDFa, HEMa, and HEKa cell pellets were collected by centrifugation, then ruptured by probe sonication and their absorption spectra was measured, and were shown in Supplementary Figure 2b. These results evidence that the cell containing photo-sensitizes molecules directly absorb photons from light and trigger photo-reactions, and also the reaction is dependent on the photon tolerance capacity of cells.

To compare the overall ECIS data with other quantitative results, the live/dead measurements were independently performed by fluorescent imaging (Figure 2). Live/dead images are shown, at different time intervals: 15, 30, 60, 80, and 150 min after each different color light exposure by on/off cycle. The overall quantitative results correlated as follows. At the 15, 30 and 60 min time after every on/off light cycle, the impedance decreased and/then recovered noticeably, while showing no changes in Live/dead cells staining. These results are consistent with the impedance curve during the intermittent light exposure, demonstrating that the ECIS method could monitor the initial onset of cellular changes. In order to quantify the number of live and dead cells as cellular functions after different color light exposure we analyzed the data and presented in Supplementary Figure 3. The quantitatively expressed data were compared with the controls, and the time dependent growth reduction was observed. Of the different color light, blue light exposure shows significantly decreased cell viability at 150 min. It has been found that around 12 percent of the cells died after a 150 min exposure. Overall high growth reductions also have been found in HDFa cells compared to control.

### WST-1 assays

Additionally, WST-1 assays were carried out to confirm the light-induced time-dependent cytotoxicity responses with a standard comparison method. For comparison of cells exposed to the different color light, the survival of skin cells was assessed by WST-1 reagent at different time points after exposure to four different color lights. As shown in Supplementary Figure 4, regardless of kinds of light employed a time-dependent decline in the survival percentage starts to appear after 60 min of exposure. However, the green color light exposure showed a significantly higher survival rate compared to other colors. Also, the blue light exposed cell shows the start of cell survival decrease of 2 - 4% (HDFa > HEKa > HEMa) at 30 min and further decrease at the level of 10 - 12 % at 150 min exposure, while no significant toxicity occurred on darkness cells until the end of the observation. Overall, the WST-1 assays revealed that blue light exposure results in the highest toxicity, followed by white light, red light, and green light. For comparison and correlation analysis, the impedance data in Figure 1 compared with the WST-1 assays. As shown in Figure 1, it is clearly evident that the measured WST-1 values do not agree with the initial impedance values upon light exposure, but after 30 min of exposure, the results are highly coincided and correlated with the WST-1 assay. These data showed that WST-1 assays can monitor the cell death as also identified by the ECIS system. However, the WST-1 assay cannot identify the initial/onset cellular changes, which is evident in the impedance curve during and after the photodynamic reaction. The minimal decrement in WST-1 values during photodynamic reaction suggested that the cell's viability was not affected even though there is a significant change in the impedance curve.

### Flow cytometry measurement

**Figure 3:**
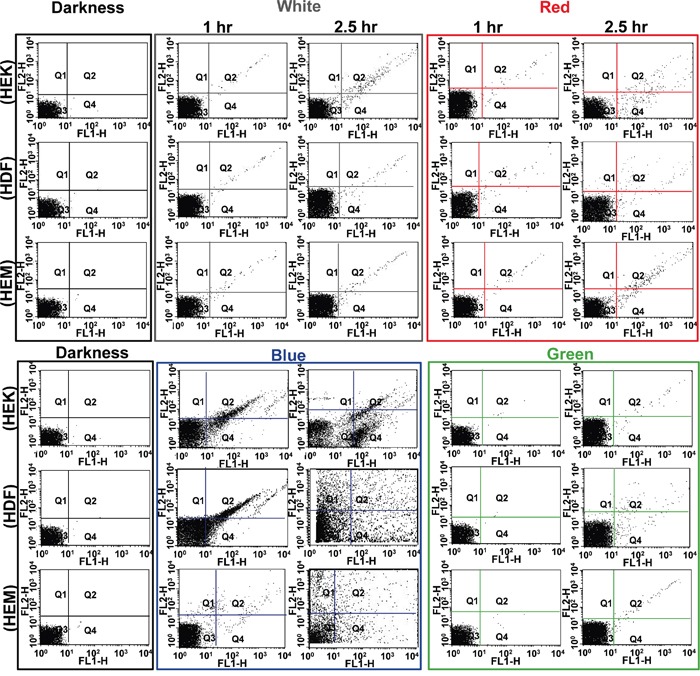
Flow cytometric analysis of effects of different color light on different skin cells Each representative scatters graphs from flow cytometry profile represents Annexin V-FITC staining in the x-axis and PI in the y-axis. Cells were stained with Annexin-V-FITC and propidium iodide. Viable cells denoted with Q3, late and early apoptotic cells denoted with Q2 and Q4 respectively, and necrotic cells denoted with Q1.

Additionally, at the end of every predetermined intermittent light On/Off cycle, the cell viability was measured by flow cytometer. The different color lights exposed cells were analyzed by fluorescence-activated cell sorting (FACS) using Annexin V-FTIC/PI staining. Annexin V-FITC and PI-stained cells were examined by cytometry (Figure 3). The dot plot profile represents the viable cells, which are denoted by Q3 (lower left quadrant) population of the cells, and late and early apoptotic cells were denoted by Q2 and Q4 (lower-right and upper-right quadrant) populations of the cells. The PI stained necrotic cells denoted by Q1 (upper left quadrant) population of the cells. It shows the different cell death behavior depending on the kind of color light. Late necrosis has been observed the most extensively in the blue exposed cell, followed by white, red and green light. These results were quite consistent with the existing results. Light exposure induced significant increments in apoptotic cell counts in early and late stages compared to the result of darkness cells (Figure 3). The quantitative apoptosis analysis of light exposed cells with phosphatidylserine on the outer side of an intact cell membrane was determined by Annexin-V-FITC/PI staining. As shown in Figure 3, early apoptosis and late apoptosis were the major mechanisms of cell demise caused by all the colored lights. At all 150 min exposures, the blue light showed significantly higher apoptosis compared to other color lights (Supplementary Figure 5), and the data were agreed with viability data. Concordant with above quantitative and qualitative findings; the blue color light exposure induces high damage in every kind of cells examined. Of the three different cells, the HDFa cells show the highest damage and followed by HEKa and HEMa cells. In particular, the percentage of viability in HEMa, HDFa and HEKa cells exposed to blue light is found to be 97.9 ± 1.0%, 95.6 ± 1.6% and 96.4 ± 0.9% respectively at 60 min exposure, and then is decreased to 94.8 ± 1.5%, 90.1 ± 1.1% and 95.9 ± 1.4% respectively after 150 min. Recently, it has been shown that light induces early apoptosis of retinal cells, which has been identified *in vitro* analysis [22].

### Cell studies by Bio-AFM

**Figure 4:**
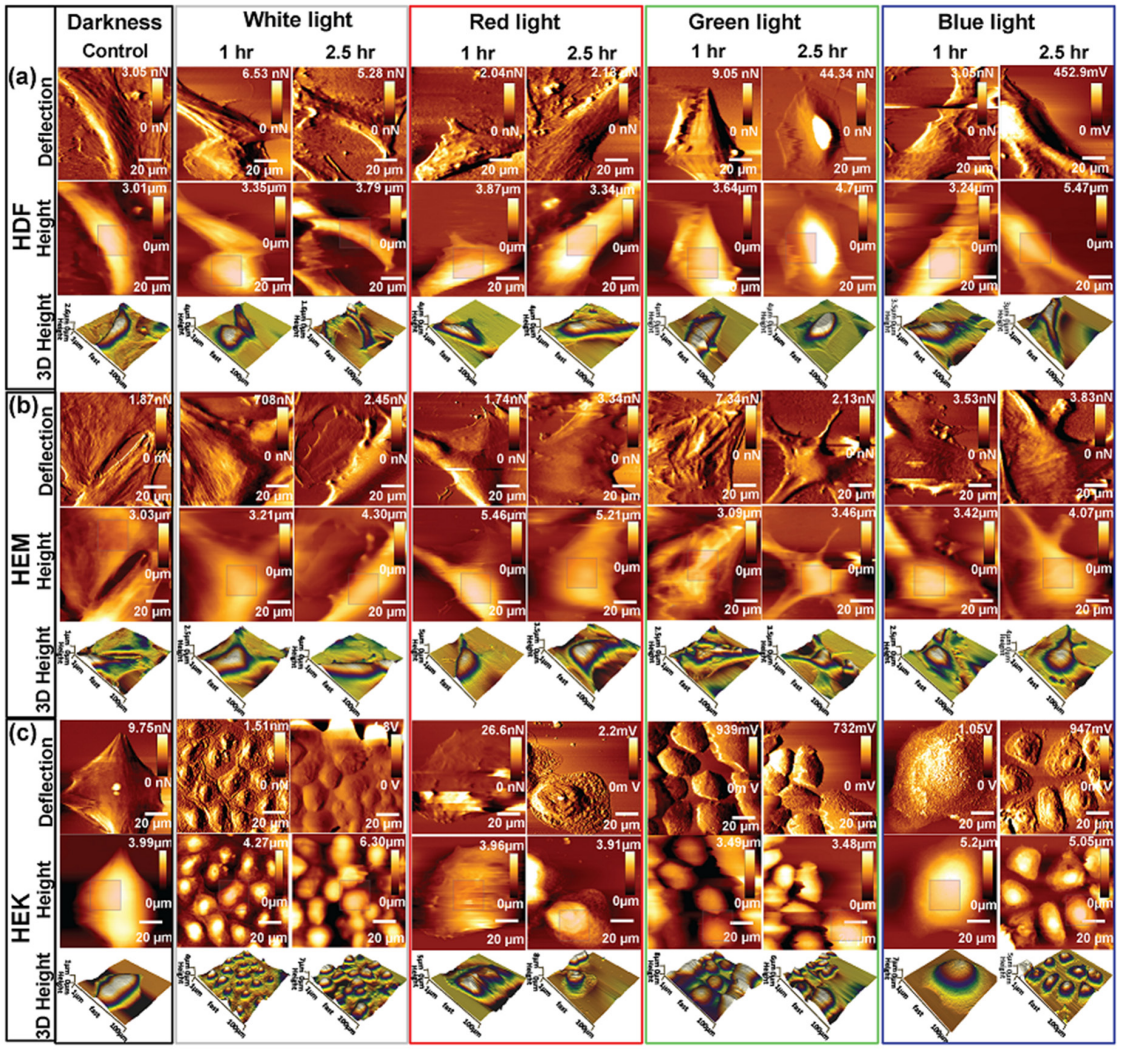
Bio-AFM images (vertical deflection, height, and 3D height) of living skin cell lines upon exposed to different color light at different time points Time-dependent changes in topographical images of **(a)** HDF cells, **(b)** HEMa cells and **(c)** HEKa cells. Cell grown in control environments shows the relatively smooth surface, whereas cells exposed to different color light showed changes in surface roughness. Scan area = 100 × 100 μm for all images.

The AFM investigations are able to provide 2D and 3D high-resolution image with detailed information about cell pathology. Any external or internal factors having an influence on cellular structures can cause the changes in mechanical properties of cells [32]. The AFM was used to analyze the effect of light exposure on the HDFa, HEMa and HEKa cells under physiological environment. Time-dependent intermittent light on/off exposures has altered cell structural organization and membrane cytoskeleton of HDFa, HEMa, and HEKa, which were clearly observable in AFM images (Figure 4). Light exposure for 60 min resulted in a significant structural reduction, and cells underwent a dramatic change in shape after a 150 min light exposure. The common surface features variations to light exposure are the increase of roughness, and the shrinks of the cell borders. In particular, the height of the HDFa and HEMa cells has increased by approximately 0.5 μm in all color light exposures (from 3.4 ± 0.3 μm of darkness cells). The height of the HEKa cells has increased by approximately 0.5 μm in blue and white color light exposure, but the green and red color light exposure does not show much change. The microtubules and actin filaments present in the cells reveal that the disassembly of the filaments occur concomitant with an increase in cell-surface area loss as increasing exposure time. The loss of the cell-surface is considered to be major changes involved in skin cell damage, which is related to the loss of basement membrane proteins, membrane-bound collagenase and surrounding other ECM protein's degenerations. These play a highly important physiological and biological regulation through the cell surrounding extracellular matrix (ECM) proteins and pigments, which are mainly responsible for offering a vast physical barrier [33] and immune network [34] and also provide a unique defense against harmful environmental. The loss of microfilament distribution in all cells at the end of the exposure regardless of color is closely related to the ECM protein's degenerations, which we largely attribute to the down-regulation of cytoskeleton integrity and increase the amount of reactive oxygen species (ROS). The de-regulation of cytoskeleton integrity may increase the risk of skin cell degeneration through direct exchange of electron or hydrogen-producing ROS [35–37]. Our results in this structural differentiation are consistent with those of previous studies regarding photochemical damage [38].

In addition to the AFM image, surface nanomechanical properties such as roughness and stiffness are important for various cellular processes [39]. In order to compare the topographical difference between the control and light exposed cells, the surface roughness values of each cell were calculated by selecting a perinuclear region of 15 × 15-μm from the AFM height images. Supplementary Figure 6a summarizes the changes in root-mean-squared roughness (Rq) of skin cells exposed to various lights. The Rq values of the darkness cells show relatively smooth surface compared to light exposed cells. On the other hand, all of the light exposed cells show obvious changes in their surface roughness; the Rq values were increased with increasing exposure except green light.

The initial green light exposure shows a decrement in roughness values, followed by the increments in roughness values with continuing exposure. All 3 cell types show a significant increment in roughness values at the end of the observation. Blue and red-light exposures resulted in relatively higher roughness compared to the white and green light exposed ones. The surface roughness data were consistent with the AFM images. The changes in topography and surface roughness of light exposed skin cells mainly caused by the increased/decreased growth of actin filaments and a pro-inflammatory response, which was consistent with the biophysical data. Further, the AFM technique was used to analysis the mechanical properties of cell surface and subsurface layers by force spectroscopy. Tip-to-cell surface interactions (indentation) were indentured 50 times repeatedly at different positions within the cell-surface region. By recording the indentation force value, the tip–cell (vertical) deflection of the cantilever was obtained and was validated using the Hertz model of the JPK software for evaluating Young's modulus of light exposed cells. The slope of the force–distance curve was estimated and was used to determine cell stiffness [40, 41]. The relative Young's modulus for darkness HDFa, HEMa, and HEKa cell was 7.0 ± 0.35, 11.3 ± 0.55, 9.1 ± 0.45 kpa, respectively. The stiffness of different color light exposed cells have been increased significantly in a time-dependent manner, except for green light-induced cells (Supplementary Figure 6b). The stiffness of green light-induced HDFa cells was 6 ± 0.35, 7 ± 0.4, 30 ± 1.5, and 34 ± 1.7 kpa in 15, 30, 60 and 150 min, respectively. During the initial light (until 30 min) exposure, it shows rather similar stiffness values of the cells that were exposed to darkness but its stiffness was increased with further exposure. A similar response was observed for all of other cells when exposed to green light. In overall, the stiffness of cells exposed to different color light exposure increased the value significantly in a similar fashion, except green light. While all colors of light resulted in stiffness increase, the blue light showed the most dramatic increase followed by red, white and green light.

Based on the above observation, it can be proposed that a very high dose of blue-light-induced notable cell damage and suppressed the cell growth, which is consistent with the biophysical and other qualitative data, and also consistent with a previous report [42]. The decrement in impedance values after light exposure suggests the development of cell damages, including cell membrane damage, DNA damage, cytoplasm damage, degeneration, and demise. This observation was consistent with results of ECIS, AFM, roughness and stiffness data. At the end of light exposure cycle, the skin cells showed a doubled stiffness value compared with that of darkness cells; this trend was directly consistent with cell membrane damage, and was also directly related to the substantial changes in intra and extra cellular behavior, including loss of basement membrane proteins, membrane-bound collagenase, cell surrounding ECM protein's degenerations, generation of ROS, inflammation, photo-oxidation, damage to the cytoplasm, deregulation of the mitochondrial membrane potential [43].

### ECM characterization by AFM

**Figure 5:**
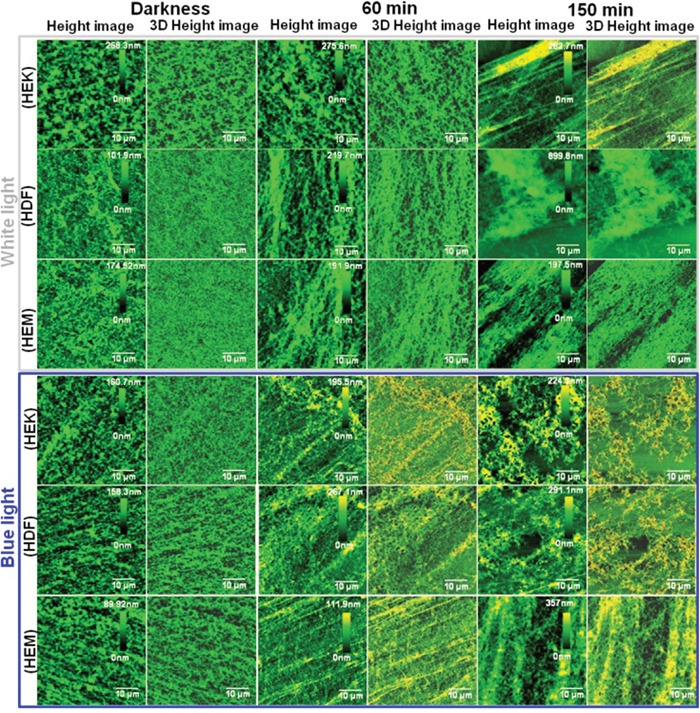
Bio-AFM assessment of human skin cell-ECM Characterization of ECM distribution in different color light-induced skin cells at different time points. Bio-AFM images (height and 3D) of skin cell-ECM reveals that the darkness cells environments show relatively smooth surface with high distribution, whereas cells exposed to different color light showed changes in roughness and distribution. Scan area = 50 × 50 μm for all images.

Among all the intra and extracellular component, the ECM is the most important component for further investigations, since ECM serves as a physical scaffold for the cellular constituents but also is coordinating cell behavior and organization. It is responsible for the extraordinary mechanical properties of many tissue type's behaviors and organizations, including skin and bone. Moreover, the ECM is produced and organized by individual cells and initiates essential biochemical and biomechanical cues that are required for various tissue functions. So it is decided to investigate the change in the ECM based mechanical function upon different color light exposure. To analyze the assembly process of ECM protein, we decellularized the ECM protein from different color light exposed confluent HDFa, HEMa, and HEKa cells. The decellularized ECM containing cover glasses were investigated for the morphological organization and biomechanical properties by Bio-AFM. The light exposures have altered the morphological organization of ECM in HDFa, HEMa, and HEKa cell as clearly shown in AFM images (Figure 5 and Supplementary Figure 7). Even though 60 min light exposure results in a minimal alteration in structural organization, 150 min light exposure has resulted in the highly altered structural organization for all cells regardless of colors of light. The common surface features of the control ECM can be summarized as having a basement membrane-like and a dermis-like matrix with uniform, dense mesh-work with very small, medium and large pores for HDFa, HEMa, and HEKa based ECM.

**Figure 6:**
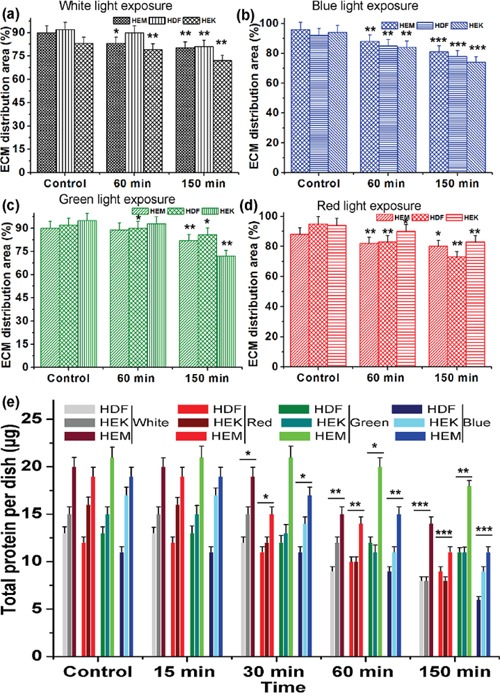
The relative distribution of ECM production by human different skin cells upon light exposure **(a-d)** Quantitative analysis of ECM distribution upon different color light exposure. **(e)** Quantitative analysis of total protein activity after exposure to different color light to different skin cell-derived ECM. Data are represented as means ± SEM. The light exposure responses were compared with control (darkness) with the level of significance set at ***P < 0.0005, **P < 0.005 and *P < 0.05.

This observation is perfectly consistent with other electron micrographs [44]. AFM images of control samples showed the typical network composed of individual ECM collagen fibrils. This kind of alteration of ECM network structures may be due to cell shrinkage during light exposure. Furthermore, the organization of collagen and elastin has altered upon different color light exposure, and the density of the extracellular matrix also has been changed. This is one of the prime inducing factors for the photo-aging process of the human skin. Moreover, the height of the HDFa and HEMa ECM has been increased under all color light exposures compare with darkness cells. However, at the same time, the distributions of ECM have been decreased severely as clearly shown in Figure 6(a-d). The shrink of ECM distribution's area is considered to be major changes involved in skin cell damage, which is related to the loss of basement membrane proteins, membrane-bound collagenase and surrounding other ECM protein's degenerations. In particular, ECM of all three cell lines shows a significant decrement in the aerial coverage. Blue-light exposures result in a relatively higher loss in coverage compared to the other light exposure and followed by red white and green light exposure. The shrinkage of ECM distribution may be related to the microtubules and actin filament fragmentation or reduction in the cell monolayers. The disassemblement of the filament, as well as shrinkage in ECM distribution, occurs with continuing exposure to the light. Additionally, the surface roughness of ECMs was measured by AFM and the surface roughness of the cells exposed to darkness is relatively low compared to light exposed cells (Supplementary Figure 8). The ECMs of light exposed cells show obvious changes in their surface roughness and the roughness values were increased with increased exposure to all light. The surface roughness data were consistent with the ECM AFM images and ECM network loss value. ECM is composed of various proteins, including collagen, elastin, laminin, fibronectin and vitronectin. Total protein levels were quantitatively analyzed via Bradford Assay. The analyzed results of protein extracts are shown in Figure 6e. A standard curve of absorbance was generated to determine the concentration of the test sample. The total protein level was higher in the darkness ECM than in light exposed ECM. The protein expression was significantly decreased with light exposure. The total protein level has exhibited a gradual loss of ECM up to end of the light exposure, during which the protein starts to degenerate or down-regulated through light-induced oxidization and/or light-induced oxidative stress. Together, these data show that protein expression was impaired in photo-induced cells of human skin. The observations from the Bradford assay indicate that the light irradiation causes highly alterations in extracellular matrix proteins and suggest that matrix degrading proteases participate in this process. In particular, the activation of matrix metalloproteinases in skin cells can also degrade other ECM protein components, including fibrin, fibronectin, proteoglycans, collagen, elastin, and laminin [45–48]. Furthermore, this activation may also induce the aging process, which has been found in skin dermal cells during ultra-violet light exposure [49]. These color lights induced activation of cellular compounds, and degeneration requires further study. Previous studies prove that both apoptosis [4] and necrosis have a certain threshold light exposure [44], thus; our data coincided well with existing results. This research obtained results give an insight that the UV-free light may sparingly contribute to aging. Moreover, it advances our understanding of the different color light-induced degenerative process. Furthermore, these biophysical and biomechanical analyses will help to develop novel therapeutic strategies to retard or cure photo-aged skin impairments.

In conclusion, the present study shows the real-time, non-invasive, label-free and onset *in vitro* photo-pathology analysis and photo-dynamic risk assessments of skin cells, which will be desirable for standard experiments. In this photo-dynamic study, we utilize the artificial light setup with an ECIS platform for the photo-pathology studies. The ECIS based bioimpedance degenerative profiling was compared with real-time bio-AFM, fluorescence microscopy, and flow cytometry techniques for analysis the status of cells under blue, green, red, white color light exposure. The initial exposure caused no changes, but at the initial exposure altered the impedance that almost completely recovered during the off cycle. The following exposure led to a greater decrease in impedance value, which was shown to be a highly tolerated dose and recovered very slowly over several hours, which further confirmed with different bio-analytical techniques. Biophysical data obtained using the ECIS systems coincides with other bio-analytical techniques. Further, cellular viability was studied and compared, and the results coincided with each other. Further, the human skin different cells, ECM, and their protein upon different color light exposures were monitored by up/down-regulation in structural, biophysical and biomechanical properties of these cells. Our experiments have shown that the dominating effect of different color lights on human skin cells is the deregulation of cell structural integrity, especially of deregulation of ECM protein. The deregulation ECM protein may be the possibility to the photo aging. The altered biophysical and biomechanics behavior might influence the normal cell functions, such as providing support, contractility, attachment, motility, cell communication, proliferation, and differentiation, which are necessary for the reform of the ECM. Taken together, these results demonstrate that the intense color light exposure can induce oxidative stress, which can produce an inflammatory response and deregulation of matrix protein in the skin, similar to the effects of UV, and therefore LED light may contribute to the signs of photo-aging or premature aging in the skin. Therefore, further studies are required to better understand the pathological processes underlying different color light-induced degeneration. These results advance our knowledge about degeneration and it fit previous theories on photo degeneration.

## MATERIALS AND METHODS

The detailed experimental procedure, including materials and reagents used, instrumentations used, HEKa, HEMa, and HDFa cell culture preparation and methods are given in the Supporting Material, under the Section I.

### ECIS experimental setup for photosensitivity analysis

The detailed photo-dynamic experimental setup which was established by our group [42, 50, 51], and comprised of ECIS system and artificial light setup was schematically described in Supplementary Figure 1. Eight LED lights were constructed in a strip with different RGB colors, eight well ten electrodes (8W10E) ECIS culture chip, thermocouples, CO_2_ incubator, LED light control setup, electric cooling fan, and PC (data-acquisition system) with a software interface. The LED strip light setup was kept inside the incubator; eight colors light constructed in a strip and were placed about 1 centimeter above the cell culture electrodes, each light was focused to each cell cultured well, and make sure that all wells received the same lighting in order to achieve the intended doses. Different artificial color lights (red, blue, green and white) were used for identifying the risk assessments of light effect on skin cells. The 8W10E ECIS chip was made-up of eight 250 μm diameters of circular gold electrodes (working electrode) and surrounded by a counter electrode for current flow. Electrodes were connected to the edge of the culture chip; the chip was plugged into the lock-in amplifier, which is connected to ECIS device and the devices were controlled by the computer. The ECIS software was utilized to control the experiments, process the data, and display the impedance values of cells. Cells were seeded into the well of the 8W10E ECIS array chip, which was covered with gold electrodes. After seeding of cells, cells are attached, spread, and proliferated on the electrodes, and thus, the impedance between the counter and working electrodes can be monitored.

For the photodynamic assay, we monitored the time-dependent different color LED light exposure effect with continuous manner. The different time points On/Off series were set from 1 min to 150 min. The parameters including wavelength, intensity, and dose of the each light were characterized by a spectroradiometer. The visible regions of the electromagnetic spectrum of each color light spectral characteristics are shown in Supplementary Figure 2a. Red, green, blue and white light wavelength exposures under a normal culture environment were studied (Supplementary Figure 2a). LED color lights having maximum peak wavelengths of 460 nm (blue), 530 nm (green), 625 nm (red) and white (400 to 700 nm) lights with stable luminous intensities (4000 mcd) at 70 mA light were used in these experiments. The irradiance energy output of the each LED at 1 centimeter was 9mW/cm-2, and they were fixed by adjusting the power. During the 1min to 150 min exposure, irradiation doses were 0.2 mJ/cm^-2^ to 300 mJ/cm^-2^. These irradiation doses were set as for 1min to 150 min exposure time, which can correlate the time dependent and dose-dependent effect.

### Experimental procedures for *in vitro* photodynamic assays

ECIS electrodes were stabilized for 10 min at room temperature using 200 mL of a 10 mM solution of cysteine, then rinsed with serum-free culture medium. The cell culture medium (without phenol red) was added to the well-containing microelectrode, then the culture chip was plugged into a lock-in amplifier and incubated at 37°C to record the background impedance value (Z0) for initial 15 min [39]. At the same time, preferred concentrations of different skin cells (HEKa, HEMa, and HDFa) were prepared from the two-day cultures. Cells were removed from culture flask using trypsin/EDTA, washed by centrifuge (3000 RPM), collected the cell pellet, then re-dispersed and counted using an automated cell counter (Countess™, Invitrogen, South Korea). Exact numbers (5 × 10^5^ cells) of cells were added to the well-containing electrode, and volume was adjusted to 400 μL using the respective phenol red free medium. Then the cells were spread, and proliferated on the electrodes after incubation. After attachments, the LED color light was exposed to the cells. Prior to light exposure, the medium was replaced with fresh medium. Additionally, a sterile wet tissue was placed near to the ECIS chip to control the excess of evaporation. The control (darkness) cell was also maintained by preventing the light exposure. The biophysical responses of cellular behavior upon light exposure were reflected in impedance signals, which are registered by the system. The photodynamic assay was monitored for 1 min to 150 min continuously with intermittent On/Off series of different color LED at different time points. Impedance data were acquired and processed using the software. The cellular activity was observed at frequencies 24,000, 16,000, and 24,000 Hz for HDF, HEM, and HEK cells, respectively. These frequencies are known to be optimum and most sensitive for individual skin cells [22, 51]. Thus, these frequencies were used for further studies. The impedance-based physical information was calculated from the average signal of the entire cells. The time point cell viability was determined by WST-1 assay, Live/Dead Staining and FACS analysis and detailed protocols were given in Supporting Material, under the Section II.

The structure, morphology, topography and biomechanics changes of different color light exposed human skin cells and their respected ECM (these are prepared by using a standard protocol, and more details see Supporting Material, under the Section III) were analyzed by Bio-AFM. After light exposure, the medium was replaced with fresh media. The culture dish was then mounted on the appropriate stable live cell culture holder of the AFM. Culture dish containing respective fresh culture media was kept at 37°C, and the images were acquired from the physiological liquid environment using soft type standard V-shaped silicon nitride gold-coated cantilevers with a nominal spring constant of 0.06 N/m to minimize cell damage. The inverted optical microscope was used to navigate the cantilever tip over the region of interest and allowed to establish a direct correlation between optical AFM images. The scan rate for AFM imaging was set at 0.7 Hz with surfaces scan size range of 100 × 100 μm. All the images were processed using a first-order plane-fit function available in the JPK processing software to eliminate tilt in the scanned image. The roughness of the each cells ECM was quantitatively analyzed from the obtained 2D height scale images (x–y scan range, 100 × 100 μm) using the bio-AFM JPK offline data processing software v3.3.25.

The biomechanical changes were measured using nanoindentation method with the soft cantilever (nominal stiffness = 0.01 N/m) with a 5 μm SiO_2_ particle attached to it for force spectroscopic analysis. Initially, the light exposed samples were imaged in the liquid contact mode to locate the cells, and the biomechanical changes were measured in ECM (Supporting Material, under the Section III, Bio-AFM structural and biomechanics assessment of human skin cells and ECM). Tip–sample deflection curve was plotted to evaluate the relative stiffness (Young's modulus) of the samples. Young's modulus was calculated using Hertz's contact mechanics model of the JPK data processing software. Finally, the total ECM proteins were measured using Bradford's reagents by following the standard protocol (Supporting Material, under the Section IV). And the statistical analyses are given in the Supporting Material, under the Section V.

## SUPPLEMENTARY MATERIALS FIGURES



## Abbreviations

ROS: reactive oxygen species
ECM: extracellular matrix
HEKa: human epidermal keratinocytes, adult
HEMa: human epidermal melanocytes, adult
HDFa: human dermal fibroblasts, adult
LED: light-emitting diode
ECIS: electric cell-substrate impedance sensing
Bio-AFM: bio- atomic force microscopy
FACS: fluorescence-activated cell sorting
EDTA: ethylenediaminetetraacetic acid
