# Supplementary material for: An ultra-sensitive biophysical risk assessment of light effect on skin cells
Source: Oncotarget. 2017 May 24;8(29):47861–75. doi: 10.18632/oncotarget.18136 (PMC5564611; doi:10.18632/oncotarget.18136)
Supplement: Supplementary file 1 [file oncotarget-08-47861-s001.pdf]

An ultra-sensitive biophysical risk assessment of light effect on skin cells

SUPPLEMENTARY MATERIALS

ELECTRONIC SUPPLEMENTARY INFORMATION (ESI)

EXPERIMENTAL SECTION

SECTION I

Materials and reagents

Alexa fluor 488, 2-(4-iodophenyl)-3-(4-nitrophenyl)-5-(2,4-disulfophenyl)-2H-tetrazolium (WST-1) reagent, bovine serum albumin, Bradford reagent, Triton X-100, 10 mM NH_4OH and trypan blue dye were purchased from Sigma Chemical Co., St. Louis, MO. Trypsin-EDTA, Dulbecco's phosphate-buffered saline (DPBS) and penicillin-streptomycin were purchased from Gibco Laboratories (Grand Island, NY). Annexin V-FITC Apoptosis Detection Kit was purchased from BD Biosciences, San Jose, CA. Live/Dead Staining kit (Biovision), and phosphate-buffered saline (PBS) buffer solutions (pH 7.4) were purchased from Invitrogen. The other chemicals used were of pharmaceutical grade. Milli-Q water (18.2 MΩ) was used throughout the experiments.

Instrumentation

This study used LED light producing blue-460 nm, green-530 nm, red-625 nm and white color light (380 to 780 nm) purchased from Hana Technology Co., Seoul, South Korea. Biophysical characteristics were studied using ECIS system; software, electrode arrays, and a lock-in amplifier were obtained from Applied Biophysics (Troy, NY, USA). The temperature was monitored and controlled by a thermocouple (K-type, Omega Engineering, Inc. Stamford, CT). Electric cooling fan for neutralizes the elevated temperature by circulating the air. Structural characterizations were studied with a conventional fluorescence microscope. Surface features and biomechanics of the cell and SEM were studied by a bio-atomic force microscopy (Bio-AFM; Nanowizard II, JPK instruments, Berlin, Germany) with the inverted optical microscope (Nikon Instruments Eclipse Ti; Amsterdam, Netherlands) in liquid contact mode. After exposure, the medium was exchanged with fresh medium and used for AFM studies.

Cell preparation

The HEKa (13 ages), HEMa (18 ages), HDFa (12 ages) cells (derived from the foreskin of men belonging to the yellow color skin) with respective growth medium containing without phenol red (human keratinocyte growth medium (CB-HEK-GM02), human melanocyte growth medium (CB-MEL-GM03), human fibroblast growth medium (CB-HF-GM02) and supplements were purchased from CEFO Ltd, Seoul, South Korea. HEKa, HEMa, HDFa cells were washed once and cultured in T-25 flasks with their own respective growth medium (phenol red free), media were supplemented with 10% FBS and 1% antibiotics solution. The T-flask were incubated at 37°C under a humidified environment of 5% CO_2. After 3 days, the non-attached cells were washed; the cell cultures were maintained in the respective growth medium for about 4 days and before getting the confluence the cells were subcultured using trypsin/EDTA. 2nd - 5th passage of mono-disperse cell suspension was used by following the standard cell culture protocol. Cell viability was measured using the trypan blue exclusion test for all the experiments.

SECTION II

Cell viability determination

WST-1 assay

In parallel, cell viability was measured using a WST-1 test kit (Roche) following the manufacturer's protocol and the viability was checked every predetermined time point. In brief, exact numbers (2×10^4) cells were seeded into each well in black colored 96-well culture plates and kept in the CO_2 incubator for 10 to 15 hrs at 37 °C prior to starting the experiment. After 90 % confluent, they were exposed to different color light with the intermittent On/Off cycle. At the end of every predetermined time of exposure, the medium in each well was replaced with 100 µL of fresh medium containing 10 µL of WST-1 reagent and incubated for 4 h at 37°C. Then they were examined using a microtiter plate reader at a wavelength of 450 nm (PerkinElmer VICTOR 3 microplate reader (Waltham, MA, USA). The output observation is directly correlated to the number of cell viability.

Live/Dead staining

Cell viability was qualitatively and quantitatively evaluated using Live/Dead Staining kit (Biovision) and was followed the manufacturer's protocol. At the end of

every predetermined time of exposure, the medium was removed from the ECIS chip, and the previously prepared staining solution was added to the wells and incubated for 15 min at 37 °C. The stained cells (live (Ex/Em 488/518 nm) and dead (Ex/Em 488/615)) were visualized by fluorescence microscopy using a band-pass filter (detects FITC and rhodamine). Similar times and conditions were set for darkness.

FACS analysis

Annexin V-FITC apoptosis detection

Additionally, the cell activity was monitored by flow cytometry as described previously [1]. While cells are exposed to a different color of lights, the activity of exposed cells is measured by flow cytometer (BD FACSCaliburTM Flow Cytometry, BD Bioscience, San Jose, CA, USA). Briefly, cells were seeded into the 24 well culture plates and kept in the CO_2 incubator at 37 °C for 10 to 15 hrs to reach 80 – 90 % confluent density. After getting confluent, the cells were exposed to different color light with the intermittent On/Off cycle. At the end of every predetermined exposure time, the cells were harvested by trypsinization, centrifuged at 1500 RPM for 5 min, washed with PBS (1X), and were collected. Then the collected 10^5 cells/mL was resuspended in a binding buffer, then 5 µL of Annexin V-FITC (conjugated with fluorescein isothiocyanate) and 5 µL of propidium iodide (PI) was added and mixed into the cell suspended solution for staining. Then the cell suspension was set aside for 30 minutes in the dark at room temperature following the manufacturer's instructions. The analysis was performed by flow cytometry with data-acquisition of 10,000 cells. The cell activity profiles were then analyzed using BD Cellquest Pro software and the apoptosis analysis follows the BD Annexin V FITC assay protocol. All experiments were carried out using the same instrument settings.

SECTION III

Bio-AFM structural and biomechanics assessment of human skin cells and ECM

Cell-surface morphology and topography analysis

AFM studies were carried out in a contact mode using a high-resolution bio-atomic force microscopy (Bio-AFM). Morphological and topographical differences between cells exposed to different color light at different time points (60, and 150 min) and darkness cells were determined by obtaining 2D and 3D bio-AFM images. After light exposure, the growth medium was replaced with fresh culture media. The culture dish was then mounted on the appropriate stable live cell culture holder of the AFM. Culture dish containing respective fresh culture media was kept at 37°C and was used for bio-AFM studies. All images were acquired in the physiological liquid environment using soft type standard V-shaped silicon nitride gold-coated cantilevers (Microlever D, Veeco, Santa Clara, CA) with a nominal spring constant of 0.06 N/m to minimize cell damage. The inverted optical microscope was used to navigate the cantilever tip over the region of interest and allowed to establish a positive correlation between optical images and AFM structural images. The scan rate for AFM imaging was set at 0.7 Hz with 512 × 512-pixel resolution with surfaces scan size range of 100 × 100 µm. All the images were processed using a first-order plane-fit function available in the JPK processing software to eliminate tilt in the scanned image.

Surface roughness analysis of different color light exposed cells

The surface roughness of the intermittent light exposed cells, and darkness cells were quantitatively analyzed from the obtained 2D height scale images (x–y scan range, 100 × 100 µm) using the bio-AFM system with the JPK offline data processing software v3.3.25. Root-mean-square value of surface roughness was analyzed from the 20 µm of selected to scan an area of different center regions of the different cells, which were exposed to different color light for 15, 30, 60, and 150 min. Surface roughness was calculated by applying a mean filter to raw or original data.

Biomechanical analysis of different color light exposed cells

Bio-AFM analysis to determine the nanomechanical changes of skin cells upon different color light exposure was performed in liquid contact mode under physiological conditions. Biomechanical changes in cells exposed to light for 60 and 150 min were measured using nanoindentation method with the soft cantilever (nominal stiffness = 0.01 N/m) with a 5 µm SiO_2 particle attached to it (Novascan, Technologies, Inc.) for force spectroscopic analysis. Initially, the light exposed cell's samples were imaged in the liquid contact mode to locate the cells. Biomechanical changes in each cell type were analyzed by scanning different positions in the perinuclear region of cytoplasm, which were selected from the contact mode image. After selecting the desired area, the cantilever was approached onto the selected region at a speed of 1 µm/s with a contact force of 1 nN. Cantilever deflection was decreased in the range of 500 nm to obtain a gentle indentation, which prevented cell membrane damages. After the induction of force on the surface, the cantilever was lifted and cantilever deflection was recorded. Tip–cell deflection curve was plotted to evaluate the relative stiffness (Young's modulus) of the cells. Young's modulus was calculated using Hertz's contact mechanics model of the JPK data processing software.

Preparation of extracellular matrix (ECM)

HEKa, HEMa, and HDFa were cultured in the respective mediums, supplemented with respective growth supplements and antibiotics. The cell was seeded at each density of 2 × 10^4/cm^2 onto the 24 well culture plates containing a cover glass (Paul Marienfeld GmbH & Co. KG's, Germany) and incubated at 37 °C with CO_2 environments for 3-4 days until getting confluence. The media were replaced with a fresh one every two days. Upon reaching the cell confluence, they were exposed to different color light with the intermittent On/Off cycle. At the end of every predetermined exposure time, the cells were treated briefly with a detergent solution containing 0.25 % Triton X-100 and 10 mM NH4OH. The samples were washed with PBS, then add PBS, which containing 50 IU/ml of DNase I and 2.5 µL/mL of RNase A and incubated at 37 °C for 1 hrs. Finally, the decellularized cover glasses containing ECM samples were gently washed with PBS. Then they were transferred for AFM and protein analysis.

Light-induced ECM assessment based on Bio-AFM

Morphological and topographical differences between ECM exposed to different color light at different time points (60, and 150 min) and control ECM was determined by observing 2D and 3D bio-AFM images. Roughness and elastic moduli were explored quantitatively with the AFM analysis. Results of the percentage of ECM protein expression and coverage's are calculated. The spatial distribution of the ECM spreading area was quantified using the ImageJ software (ImageJ, National Institutes of Health (NIH), Bethesda, MD). In the image-processing, the color contrast of each AFM ECM image was enhanced using the color display option to obtain ECM distribution expression around the 100 × 100 µm sized cover glass. After adjusting the threshold to identify the ECM coverage area, the ECM spreading area was obtained. The extracellular molecule expression in cells has been quantitatively measured. Results of the percentage of ECM protein expression and coverage are calculated, and the mean and standard error of these values were calculated.

SECTION IV

Protein determination

The total protein concentration in the cells with different exposure history was quantitatively measured by using Bradford's reagent and bovine serum albumin (BSA) as the standard [2]. Total protein in the light exposed cells and in darkness cells were estimated at two-time points (60, and 150 min). Cultured cells with and without light exposure were treated with 0.5 % Triton X-100 solution, scraped, collected, and vortexed for 30 min. For protein standards, 0, 0.5, 1.0, 1.5, 2.0 and 2.5 mg/mL of BSA protein was prepared in DPBS buffer. The 5 µL each of BSA and light exposed samples were added to each well of a 96 well microtiter plate, followed by the addition of 250 µL of Bradford's reagent. It was then mixed well and kept aside for 30min at room temperature. The absorbance was measured by using a microplate reader at 595 nm (VICTOR 3TM Multilabel Counter, model 1420–032, Perkin Elmer, Waltham, MA, USA). The amount of total protein present in the samples with and without light exposure was calculated from the standard curve using the following formula. Light exposed test protein (mg/mL) = (Test absorbance/Standard absorbance) × Concentration of the standard (mg/ml).

SECTION V

Statistical analysis

All the data were analyzed by Student's t test using Microsoft Office Excel 2010 Statistical Data Analysis Tool and were expressed as a mean ± standard deviation. All the experiments were conducted in triplicate, and the results were analyzed, and compared with the corresponding control experiments (without light exposure, darkness) and the level of significance set at ***$P < 0.0005$, **$P < 0.005$, and *$P < 0.05$.

REFERENCES

1. Zakaria AB, Picaud F, Rattier T, Pudlo M, Dufour F, Saviot L, Chassagnon R, Lherminier J, Gharbi T, Micheau O, Herlem G. Nanovectorization of TRAIL with single wall carbon nanotubes enhances tumor cell killing. Nano Lett. 2015; 15:891-895.
2. Bradford MM. A rapid and sensitive method for the quantitation of microgram quantities of protein utilizing the principle of protein-dye binding. Anal Biochem. 1976; 72: 248-254.

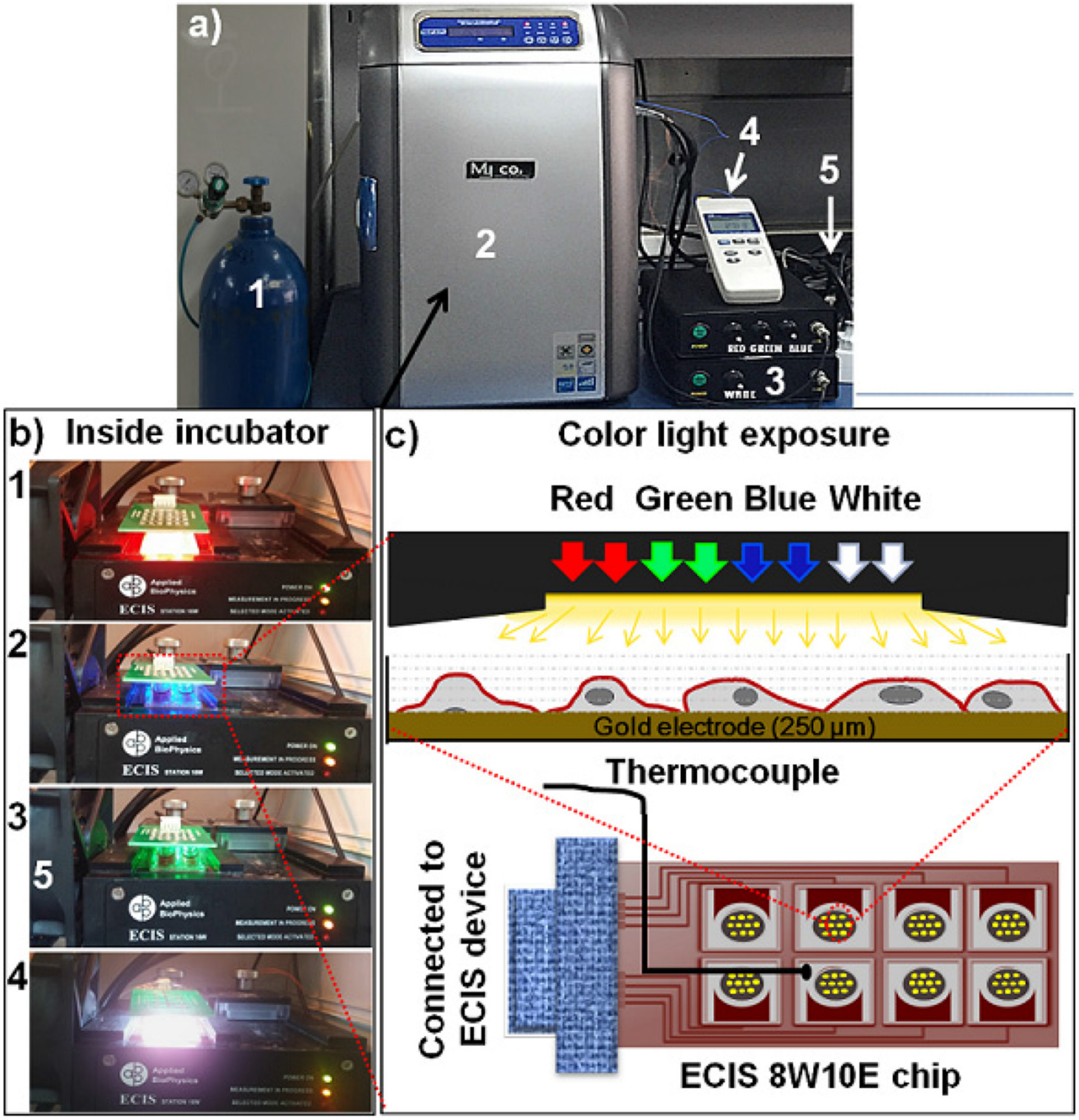

Supplementary Figure 1: A platform based on ECIS technique was constructed for analyzing light-cell interaction's studies **(a)** and the details of ECIS device with *in-vitro* LED light exposure setup **(b)** and the details of 8W10E ECIS chip with light exposure setup **(c)**. 1) CO_2, (2) incubator, (3) different color LED light power control setup, (4) thermocouples, (5) electric cooling fan control **(b)** The light setup inside the incubator, eight LED color light constructed in a strip and was placed above the 8W10E ECIS chip, which was focusing on each cell cultured well. 1) Red-light exposure, (2) blue-light exposure, (3) green light exposure, (4) white-light exposure and (5) cooling fan for circulating the air. **(c)** The details of an 8W10E ECIS chip with gold electrodes, diameter 250 µm. Cells were seeded in the well of the 8W10E ECIS chip, which was covered with gold electrodes. The chip was connected to the lock-in amplifier and was controlled by ECIS device and computer. After cell's seed, cells are attached, spread, and proliferated on the electrodes, and thus, the impedance between the counter and working electrodes can be detected.

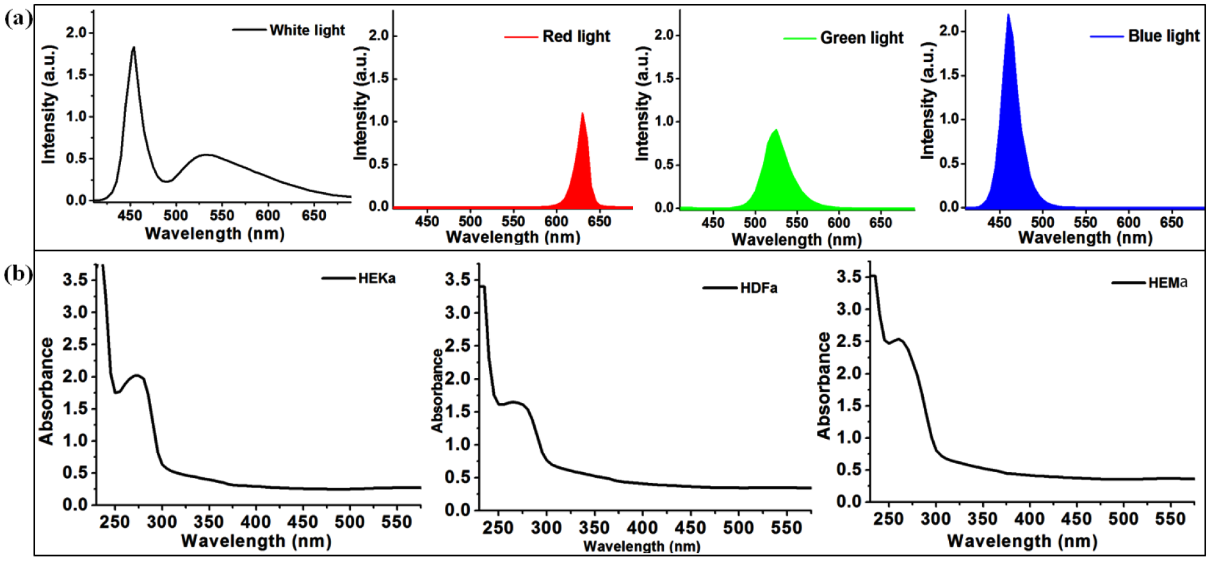

Supplementary Figure 2: LED Light source characterization **(a)** and total chromophores content evaluation **(b)**. **(a)** Each color light spectral characterization was measured by a spectroradiometer (CS-1000, Minolta). The respective light color was used in this study was within the visible region of the electromagnetic spectrum. **(b)** The absorption spectra of corresponding cells, and shows variations in total concentration of chromophores in each cell line.

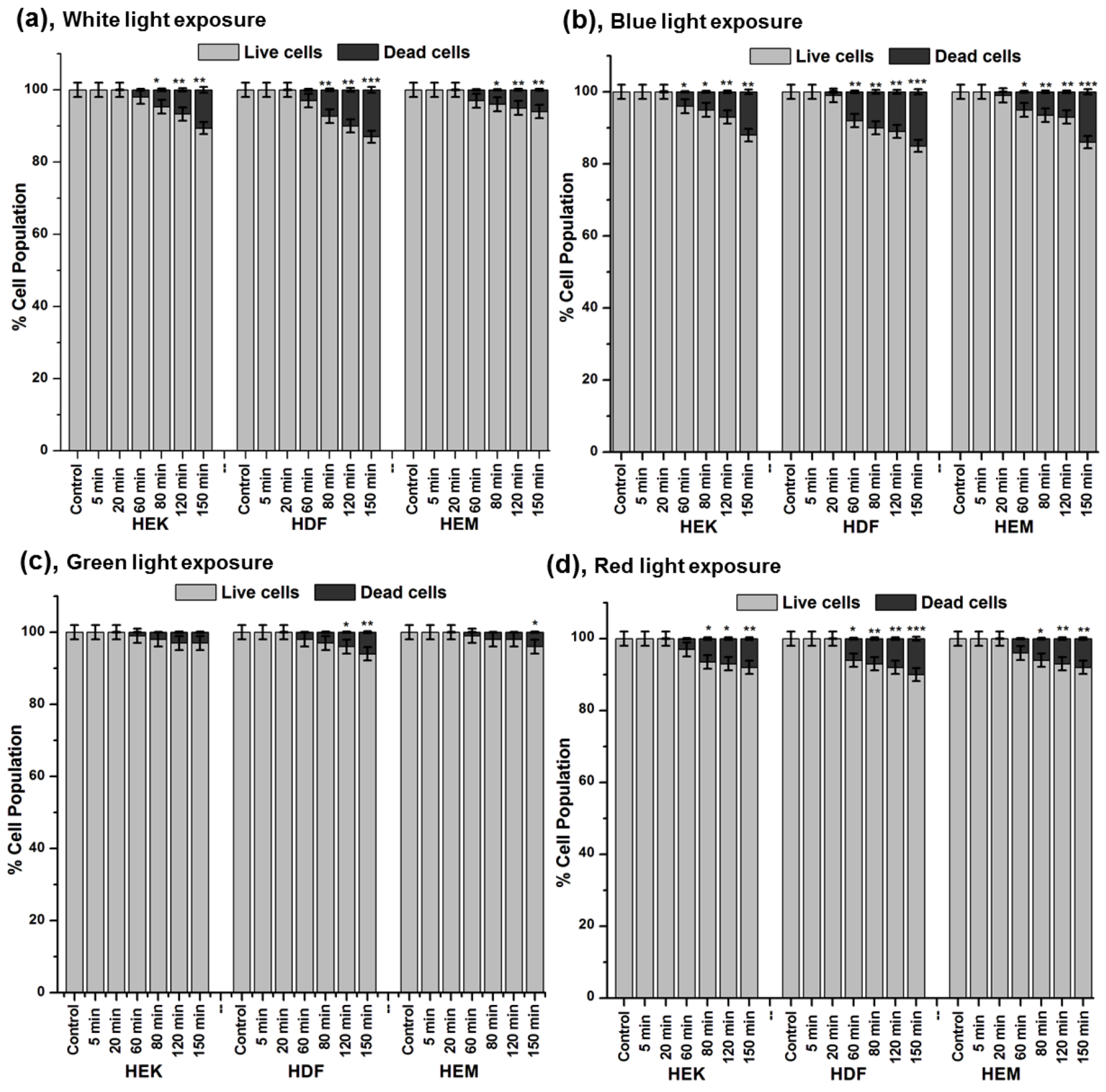

Supplementary Figure 3: Effect of different color light radiation on percentage of live and dead cells. Cells were quantified after 5, 20, 60, 80 and 150 min treatment with light. Data are represented as means ± SEM. The light exposure responses were compared with control with the level of significance set at ***P < 0.0005, **P < 0.005 and *P < 0.05.

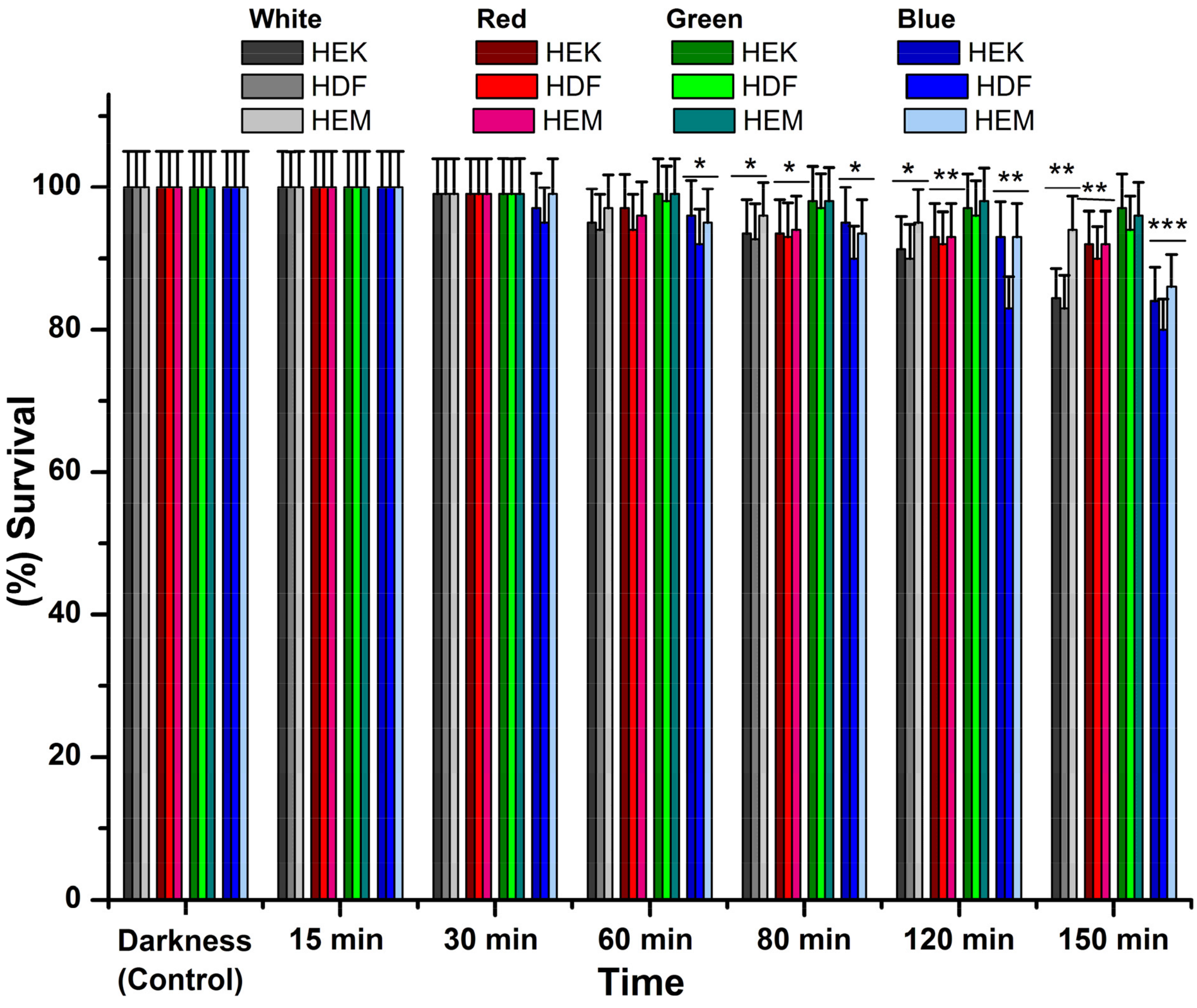

Supplementary Figure 4: WST1 assay for cell viability. Time-dependent viability changes in different skin cell upon exposure to different color light stress. Cell viability (WST1 assay demonstrating mitochondrial activity of cells after different exposure time points) was determined after 15, 30, 60, 80 and 150 min treatment with light. Data are represented as means ± SEM. The light exposure responses were compared with darkness with the level of significance set at ***P < 0.0005, **P < 0.005 and *P < 0.05.

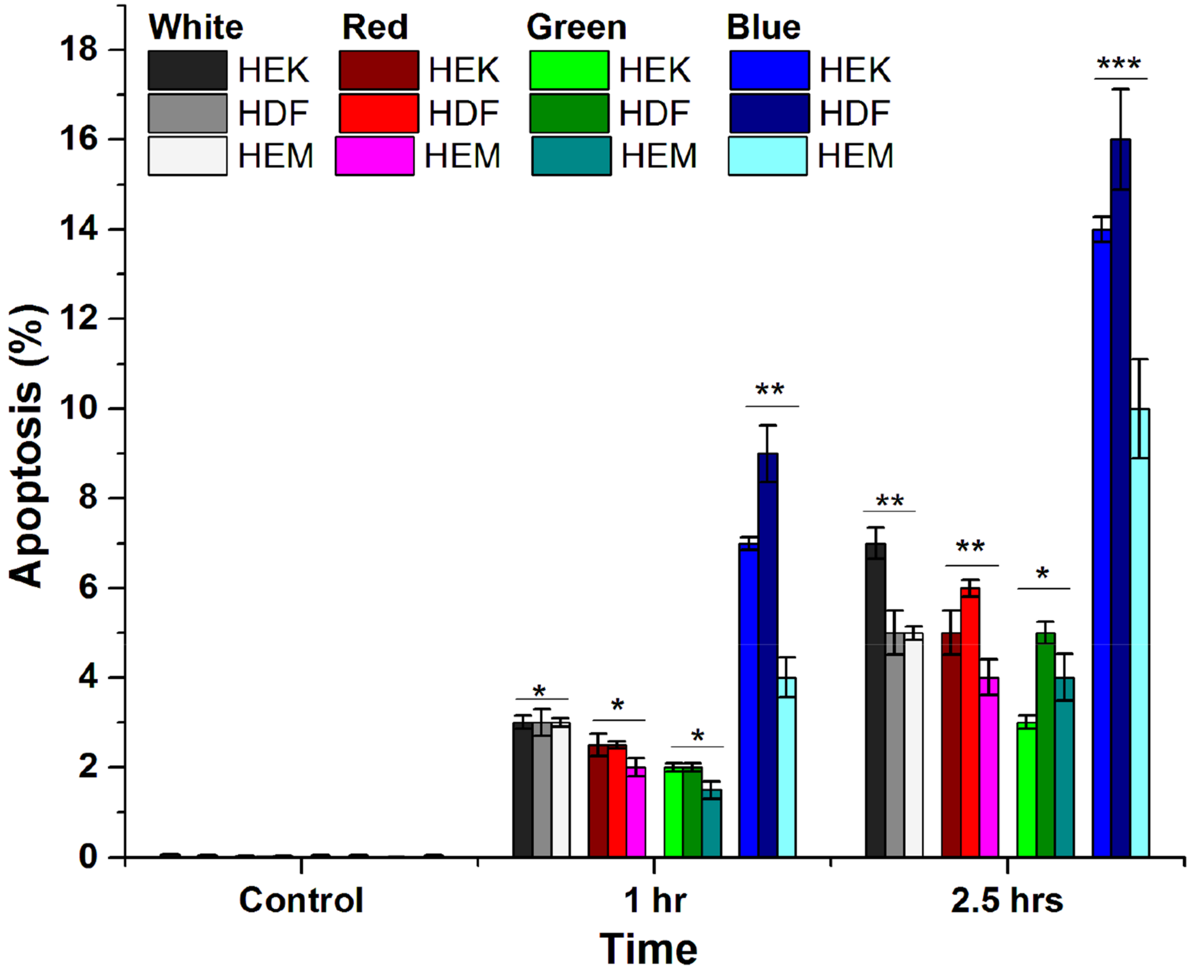

Supplementary Figure 5: Apoptosis assay. Cell death was analyzed for 60 min and 150 min by using annexin V/PI staining by FACS analysis. Apoptotic cell were calculated (early apoptosis; Annexin V^+/PI^- and late apoptosis; Annexin V^+/PI^+). Data are represented as the mean ± S.D. The light exposure responses were compared with control with the level of significance set at ***$P < 0.0005$, **$P < 0.005$ and *$P < 0.05$.

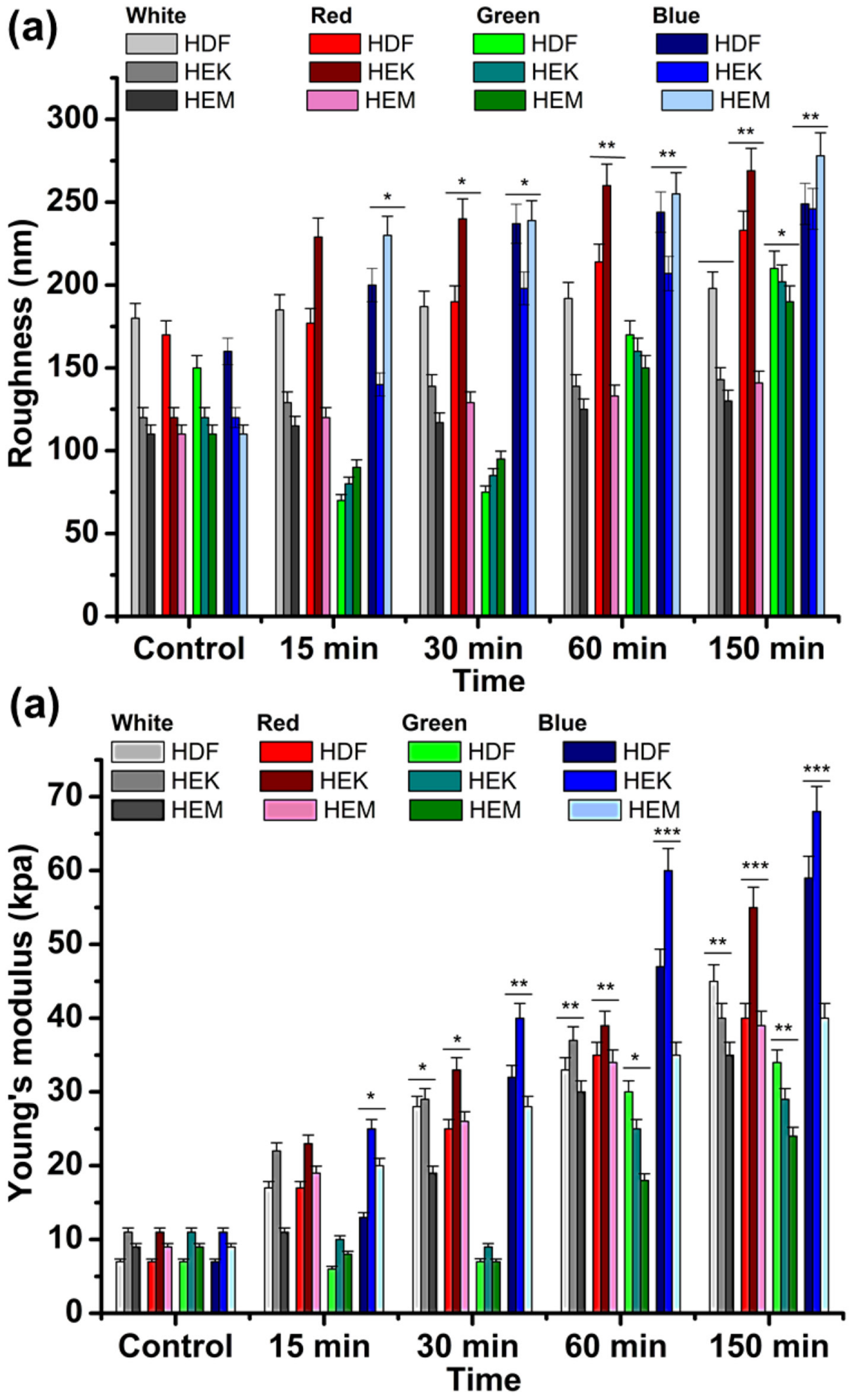

Supplementary Figure 6: Surface roughness and young's modulus of different skin cells treated with different color light. **(a)** Graph depicts the roughness value changes upon light exposure. **(b)** Graph depicts the stiffness value changes upon light exposure. The scanning area for roughness analysis was 20 µm in different cytoplasmic regions of the cells, and the stiffness was measured in the perinuclear region of the different cells. Data are represented as means ± SEM. The light exposure responses were compared with control (darkness) with the level of significance set at ***P < 0.0005, **P < 0.005 and *P < 0.05.

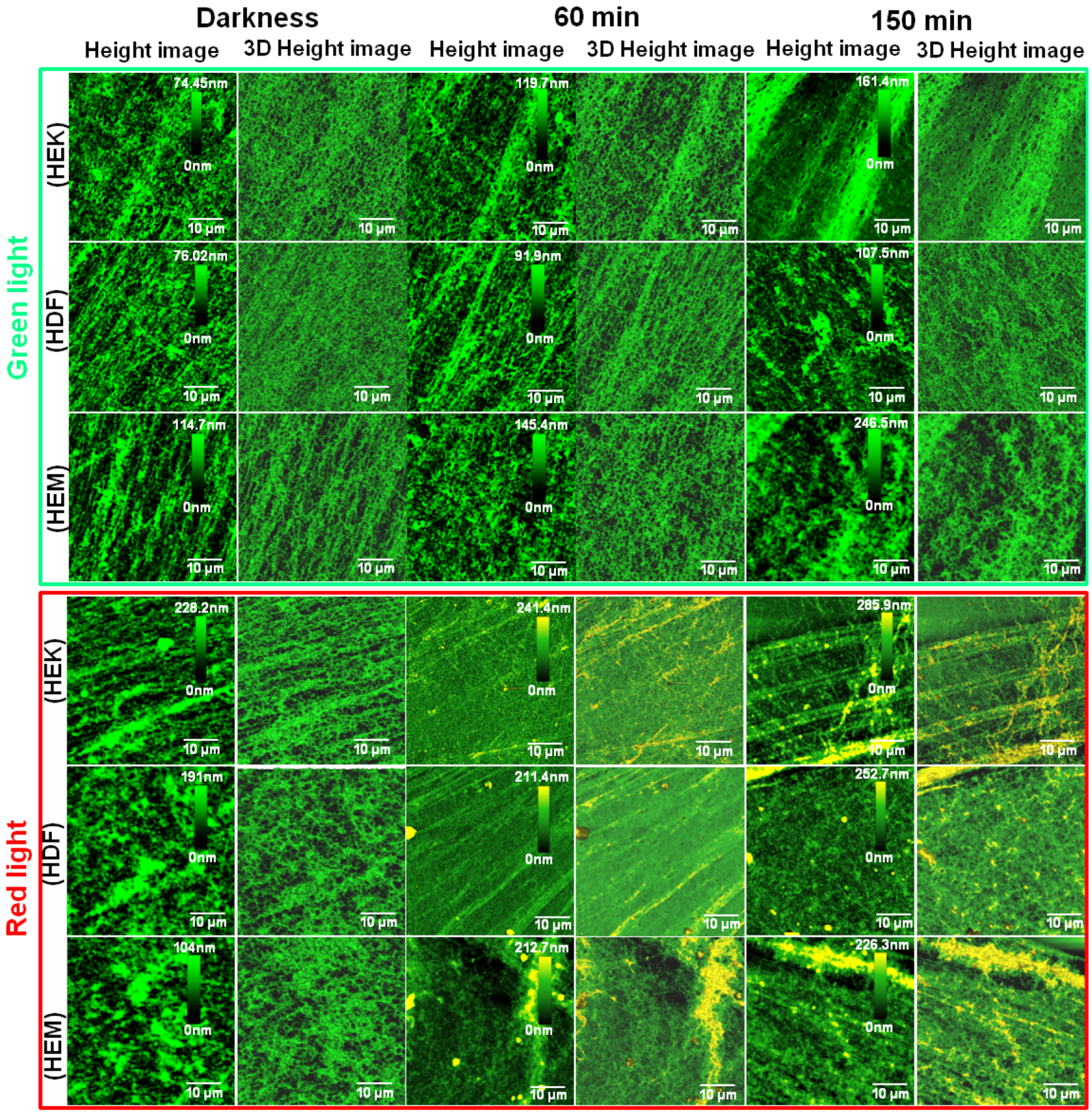

Supplementary Figure 7: Bio-AFM assessment of human skin cell ECMs. Characterization of ECM distribution in different color light-induced skin cells at different time points. Bio-AFM images (height and 3D) of skin cell-ECM reveals that the cell is grown in darkness environments show relatively smooth surface with high distribution, whereas cells exposed to different color light showed changes in roughness and distribution. Scan area = 50 × 50 µm for all images.

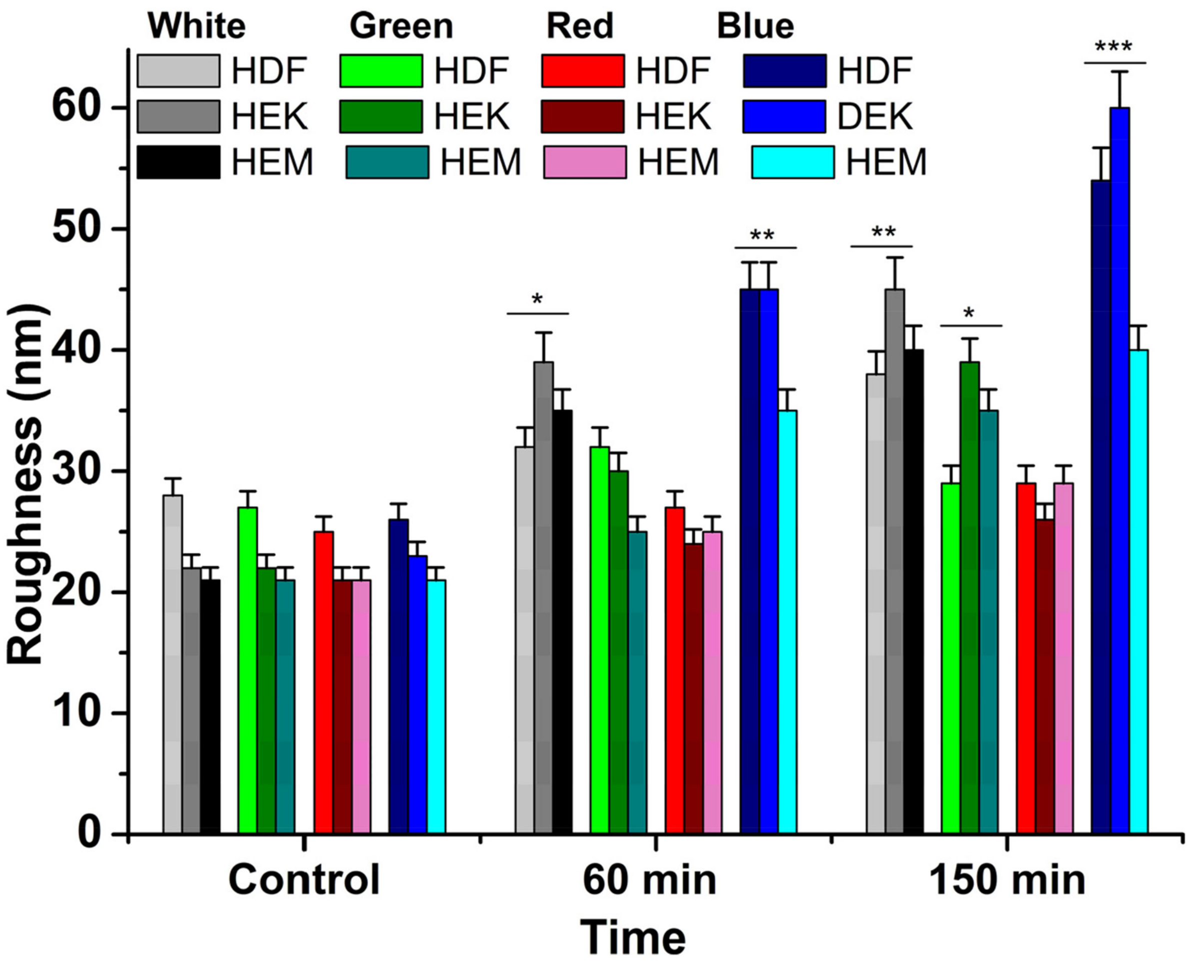

Supplementary Figure 8: Bio-AFM based surface roughness assessment of human skin cell-ECM. The graph depicts the roughness value changes upon different color light exposure. Data are represented as means ± SEM. The light exposure responses were compared with control (darkness) with the level of significance set at ***$P < 0.0005$, **$P < 0.005$ and *$P < 0.05$.
